# Editorial: Genetics of Paroxysmal Movement Disorders

**DOI:** 10.3389/fneur.2021.752000

**Published:** 2021-09-28

**Authors:** Anna De Rosa, Bettina Balint, Kishore Raj Kumar

**Affiliations:** ^1^Department of Neurosciences and Reproductive and Odontostomatological Sciences, Federico II University, Naples, Italy; ^2^Department of Neurology, University Hospital Heidelberg, Heidelberg, Germany; ^3^Department of Neurology, University Hospital Zurich, University of Zurich, Zurich, Switzerland; ^4^Kinghorn Centre for Clinical Genomics, Garvan Institute of Medical Research, Darlinghurst, NSW, Australia; ^5^Molecular Medicine Laboratory and Neurology Department, Concord Repatriation General Hospital, The University of Sydney, Sydney, NSW, Australia

**Keywords:** paroxysmal movement disorders, genetics, next generation sequencing, episodic ataxia, diagnostic and genetic algorithm

Paroxysmal movement disorders represent a heterogeneous group of rare neurological conditions, characterized by episodic and transient occurrence of hyperkinetic movements, such as chorea, dystonia, ballism, and/or ataxia, and usually normal interictal examination. These may be classified according to triggering factors, pathophysiological mechanisms, and genetic causes. In the last decades, the progress of molecular genetics has improved our knowledge of the paroxysmal dyskinesias (PxD) etiology, providing further advances in understanding the underlying pathophysiology and definition of different clinical syndromes (1, 2). High-throughput sequencing techniques like Next Generations Sequencing (NGS) have allowed us to identify several monogenic forms of PxD and a number of loci and variants playing a role in mild to strong risk factors. Genetic heterogeneity is often associated with clinical variability and complex phenotypes, leading to a significant diagnostic delay. Therefore, most neurologists are still unable to recognize these rare and complex disorders, which are often misclassified as epileptic or functional conditions and not properly and promptly treated. Furthermore, the accurate identification of these disorders is necessary for a correct family counseling.

The present Research Topic focuses on a modern revision of the last genetic discoveries in the PxD area, including four articles and four reviews aiming to advance some of the research issues that still need to be addressed.

Harvey et al. conducted an updated overview on PxD genetics based on a modern classification and reorganization, according to the underlying pathophysiologic mechanism and involved genes. Interestingly, the authors devised a detailed and useful table containing data concerning inheritance, phenotypic manifestations, association with epilepsy, and recommended therapeutic strategies for each gene. They proposed a diagnostic genetic algorithm suggesting which the first- and second-tier tests are and discussed the advantages and limitations of the latest generation techniques such as whole-exome and whole-genome sequencing.

In their review, Danti et al. dealt with the clinical, genetic, pathophysiological, and therapeutic features of paroxysmal exercise-induced syndromes occurring in childhood, focusing, in particular, on the different etiologies. The authors discussed other pediatric neurological conditions triggered by physical activity or exercise, such as myalgia, cramping, rhabdomyolysis, myotonia, stiffness, and weakness due to neurometabolic, neuromuscular, neurodegenerative, and epileptic disorders. Furthermore, they underlined the possible psychogenic etiology of these disorders, which is rarely described and recognized in childhood. Finally, the review provides a detailed diagnostic algorithm including imaging tools, metabolic and biochemical screening, and genetic testing.

Paroxysmal kinesigenic dyskinesia (PKD) is usually caused by *PRRT2* gene mutations. In their timely review, Landolfi et al. dissected the variations of the core phenotype of PKD and epilepsy due to *PRRT2* mutations and discussed rarer associations like paroxysmal hypnogenic dyskinesias, episodic ataxia, or hemiplegic migraine. They also examine the molecular and neurophysiological effects of *PRRT2* mutations and how the pathophysiological insights lead to the evolving concept of synaptopathies.

Four articles and one mini review included in this issue are focused on expanding the phenotypes and exploring the genotype-phenotype correlations of other genes variants more rarely involved in the PxD pathogenesis.

Yang et al. reported 11 patients presenting with epilepsy and developmental delay, and carrying likely pathogenic *GNAO1* gene variants, 6 of whom to be considered novel. Five cases complained of paroxysmal brief focal or truncal dystonia triggered by sound stimulus or emotional disturbances.

Kegele et al. reported two novel pathogenic variants in *KCNA1* gene, encoding a potassium channel subunit, in one case of PKD and one of non-kinesigenic dyskinesia, respectively. *KCNA1* gene mutations usually account for other paroxysmal neurological conditions as episodic ataxia type 1 (EA1), epilepsy, and other rare disorders, as isolated neuromyotonia, myokymia, and hypomagnesemia (3). The authors confirmed the heterogeneity of *KCNA1*-related phenotypes and proposed acetazolamide as first-line (in analogy with EA1) or alternative treatment to sodium channel blockers in resistant PxD patients. Furthermore, Kegele et al. also described exercise-induced dyskinesia in other two patients carrying likely pathogenic *SLC2A1* gene variants, one of which was described for the first time. Both cases also showed other clinical features that are usually observed in *GLUT1* syndrome, such as epilepsy, and/or psychomotor slowing, and/or hemiplegic migraine.

One of the phenotypes of *KCNA1* gene, EA1, is clinically characterized by attacks lasting seconds to minutes with variable symptoms and persistent myokymia (Lauxmann et al.). There is no standardized treatment approach; some patients are treated with acetazolamide, others with sodium channel blockers. Lauxman et al. reviewed this condition, used a predictive scoring system to evaluate the effects of mutations on neuronal excitability, and assessed the effects of sodium channel blockers. They found that phenytoin, carbamazepine, and riluzole may partly ameliorate the detrimental effects on channel function, supporting their use as “targeted therapies” for this condition.

*CACNA1A* gene mutations were first found to cause both familial hemiplegic migraine and episodic ataxia type 2 (4). Later, a CAG repeat expansion in *CACNA1A* was found to be the cause of SCA6 (5). Since then, our knowledge of *CACNA1A*-related disorders has rapidly expanded, as highlighted by a concise and topical review by Indelicato and Boesch. Age-dependent phenotypes are described including neuropsychiatric disorders, paroxysmal dystonia, epilepsy, and complex phenotypes combining early developmental delay and epileptic encephalopathy. Additionally, the authors reviewed the underlying pathophysiology and treatment implications and provided recommendations on genetic testing for *CACNA1A*.

Another rare paroxysmal movement disorder is represented by the alternating hemiplegia (AHC) of childhood, which is characterized by onset in infancy or early childhood of episodes of hemiplegia on either side and other paroxysmal manifestations such as dystonia, quadriparesis, seizure-like episodes, oculomotor abnormalities, and autonomic dysfunction (6). Previous studies have suggested that 85% of cases are due to heterozygous mutations in the *ATP1A3* gene (7). In a study of AHC, Cordani et al. recruited 39 patients and detected *ATP1A3* gene mutations in a higher than expected 92.3% of cases. Furthermore, they also conducted a genotype-phenotype correlation analysis.

In conclusion, this Research Topic aims to reorganize the knowledge derived from NGS technique application to PxD, emphasizing the broad clinical variability, the intersection between different phenotypes, and, finally, the importance of correctly interpreting genetic testing results. The issue also attempts to provide algorithms for identification and diagnosis, and suggests possible therapeutic strategies.

## Author Contributions

AD contributed to conception and design of the Research Topic. AD, BB, and KK collaborated to enroll potential contributors, edited the submitted abstract/articles, and wrote sections of the manuscript. All authors contributed to manuscript revision, read, and approved the submitted version.

## Conflict of Interest

The authors declare that the research was conducted in the absence of any commercial or financial relationships that could be construed as a potential conflict of interest.

## Publisher's Note

All claims expressed in this article are solely those of the authors and do not necessarily represent those of their affiliated organizations, or those of the publisher, the editors and the reviewers. Any product that may be evaluated in this article, or claim that may be made by its manufacturer, is not guaranteed or endorsed by the publisher.

## References

[1] Xu Z Lim C Tan LCS Tan EK. Paroxysmal movement disorders: recent advances. Curr Neurol Neurosci Rep. (2019) 19:48. 10.1007/s11910-019-0958-3 31187296

[2] Garone G Capuano A Travaglini L Graziola F Stregapede F Zanni G. Clinical and genetic overview of paroxysmal movement disorders and episodic ataxias. Int J Mol Sci. (2020) 21:3603. 10.3390/ijms21103603 32443735 PMC7279391

[3] de Gusmão CM Garcia L Mikati MA Su S Silveira-Moriyama L. Paroxysmal genetic movement disorders and epilepsy. Front Neurol. (2021) 12:648031. 10.3389/fneur.2021.648031 33833732 PMC8021799

[4] Ophoff RA Terwindt GM Vergouwe MN van Eijk R Oefner PJ Hoffman SM. Familial hemiplegic migraine and episodic ataxia type-2 are caused by mutations in the Ca2+ channel gene CACNL1A4. Cell. (1996) 87:543–52. 10.1016/S0092-8674(00)81373-2 8898206

[5] Zhuchenko O Bailey J Bonnen P Ashizawa T Stockton DW Amos C. Autosomal dominant cerebellar ataxia (SCA6) associated with small polyglutamine expansions in the alpha 1A-voltage-dependent calcium channel. Nat Genet. (1997) 15:62–9. 10.1038/ng0197-62 8988170

[6] Debopam S. Management of alternating hemiplegia of childhood: a review. Pediatr Neurol. (2020) 103:12–20. 10.1016/j.pediatrneurol.2019.10.003 31836335

[7] Panagiotakaki E De Grandis E Stagnaro M Erin L Heinzen L Fons C. Clinical profile of patients with ATP1A3 mutations in alternating hemiplegia of childhood-a study of 155 patients. Orphanet J Rare Dis. (2015) 10:123. 10.1186/s13023-015-0335-5 26410222 PMC4583741

